# The development of Music in Dementia Assessment Scales (MiDAS)

**DOI:** 10.1080/08098131.2014.907333

**Published:** 2014-05-16

**Authors:** Orii McDermott, Martin Orrell, Hanne Mette Ridder

**Affiliations:** ^a^ Division of Psychiatry, Faculty of Brain Sciences, University College London, London, UK; ^b^ Doctoral Programme in Music Therapy, Institute for Communication and Psychology, Aalborg University, Aalborg Øst, Denmark; ^c^ Central and North West London NHS Foundation Trust, St Charles Hospital, London, UK; ^d^ Research and Development, North East London NHS Foundation Trust, Goodmayes Hospital, Essex, UK

**Keywords:** music therapy, dementia, outcome measure, assessment

## Abstract

There is a need to develop an outcome measure specific to music therapy in dementia that reflects a holistic picture of the therapy process and outcome. This study aimed to develop a clinically relevant and scientifically robust music therapy outcome measure incorporating the values and views of people with dementia. Focus groups and interviews were conducted to obtain qualitative data on what music meant to people with dementia and the observed effects of music. Expert and peer consultations were conducted at each stage of the measure development to maximise its content validity. The new measure was field-tested by clinicians in a care home. Feedback from the clinicians and music therapy experts were incorporated during the review and refinement process of the measure. A review of the existing literature, the experiential results and the consensus process enabled the development of the new outcome measure “Music in Dementia Assessment Scales (MiDAS)”. Analysis of the qualitative data identified five key areas of the impact of music on people with dementia and they were transformed as the five Visual Analogue Scale (VAS) items: levels of *Interest, Response, Initiation, Involvement* and *Enjoyment.* MiDAS comprises the five VAS items and a supplementary checklist of notable positive and negative reactions from the individual. This study demonstrates that it is possible to design and develop an easy to apply and rigorous quantitative outcome measure which has a high level of clinical relevance for people with dementia, care home staff and music therapists.

## Introduction

Evaluation of music therapy is a complex task. Quantitative and mixed methods studies require outcome measures to evaluate the impact of music therapy. However, there are concerns that clinically significant changes are often highly individual, and standardised outcome measures may not always portray what matters most to the client.

A large number of quantitative studies on the effect of music therapy on people with dementia focus on the reduction of psychiatric symptoms (e.g., Brotons & Marti, 2003; Ledger & Baker, 2007, 2010; Raglio et al., 2008, 2010; Svansdottir & Snaedal, 2006). Well-established psychiatric measures such as Neuropsychiatric Inventory (NPI) (Cummings et al., 1997), Cohen-Mansfield Agitation Inventory (CMAI) (Cohen-Mansfield, 1986) and various depression scales (e.g., Geriatric Depression Scale, Hamilton Depression Rating Scale, Cornell Scale for Depression) are frequently used. Evidence for short-term reductions in behavioural disturbances and improved mood are consistent, but no long-term effect of music therapy has been reported in current literature. This is partially to do with study design since the lengths of the intervention and follow-up period tend to be short- to medium term as noted in recent reviews (e.g., Livingston, Johnston, Katona, Paton & Lyketos, 2005; Vink, Bruinsma & Scholten, 2013a). Ledger and Baker (2007) investigated the long-term effects of music therapy on agitation in nursing home residents, but found no differences between the intervention group and the control group “in the range, frequency and severity of agitated behaviours manifested over time” (Ledger & Baker, 2007). This raises questions whether (1) music therapy is not an effective intervention for long-term reduction of behavioural disturbance, or (2) the outcome measures used in music therapy studies need to be reconsidered.

Our narrative synthesis review (McDermott, Crellin, Ridder & Orrell, 2013) found no studies that used dementia-specific validated music therapy outcome measures. Some researchers had devised their own non-validated measures (e.g., Ashida, 2000; Brotons & Marti, 2003) or adapted an outcome measure originally developed for music therapy with children with pervasive developmental disorder (Raglio et al., 2008). A “music therapy assessment tool” was developed to evaluate behavioural responses of people with Alzheimer’s disease (Glynn, 1992) when they were “exposed to 30 minutes of selected music, which was delivered through the use of cassette tapes”. Although the study claims to have a high inter-rater reliability (*r *= .970−.999), the tool to assess responses to listening to “taped music” which can be delivered by nurses (Glynn, 1992) is not appropriate to evaluate music therapy where the interventions are delivered by trained music therapists using defined therapeutic models. Two dementia-specific music therapy outcome measures – the “Residual Music Skills Test (RMST)” (York, 2000) and the “Music Performance Tasks” (MPT) (Lipe, 1995) – were constructed to investigate musical reactions and responses of people with dementia during musical tasks. Both measures have undergone some psychometric testing and reported a high test–retest reliability (RMST *r *= .9168 *p *< .001) and a high internal consistency (MPT α = .85–.95). While measuring a client’s musical responses can be useful, the RMST and the MPT may not be suitable outcome measures for studying the clinical effectiveness of music therapy and the well-being of an individual.

There is a need to develop a psychometrically validated music therapy outcome measure that reflects a holistic picture of therapy outcomes including increased positive responses of people with dementia. This need was also acknowledged in a recent research article by Vink et al. (2013b) who have noted that “in relatively few studies, a possible increase in positive behaviours is addressed. There are still few validated outcome measures available for this purpose. More research studying the effect of music therapy on aspects of positive wellbeing is welcomed”.

No standardised quantitative outcome measure may ever fully capture meaningful musical experiences of individuals. Nevertheless, there is a need to understand and measure what people with dementia themselves would consider important in music therapy rather than only focusing on the impact of music therapy on behavioural and psychological disturbances. The new outcome measure needs to be developed based on the clinical relevance and musical experiences of people with dementia.

## Aims

- To investigate what aspects of musical experience people with dementia value and identify what may be clinically meaningful to measure
- To develop an observational outcome measure for music therapy with people with moderate to severe dementia
- To pilot and field-test the outcome measure in practice

## Method

### Design

**Figure 1.**
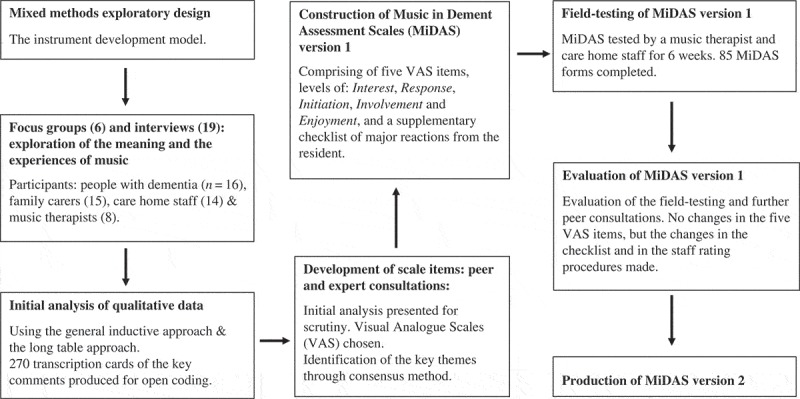
Summary of the MiDAS development.

Mixed methods designs are popular in music therapy research as they enable the generalisation of results and the understanding of “phenomena and complex therapeutic interactive processes in real-world settings” (Ridder, 2013). The ultimate goal of this study is to develop a rigorous, clinically relevant quantitative music therapy outcome measure from qualitative data. This study utilised the “mixed methods exploratory design: the instrument developmental model” (Creswell & Plano Clark, 2007). Qualitative data were obtained through focus groups and interviews, followed by open coding and thematic analysis of the data. Expert and peer consultations were conducted during the data transformation and the theory development process, and further data reduction and the finalisation of scale items were also supported through the consensus method. The new outcome measure was field-tested by clinicians in a care home. Feedback from the clinicians and external music therapy experts were incorporated during the review period of the measure. Finally, the revised outcome measure was produced after the researchers achieved consensus. Figure 1 shows a summary of the development of MiDAS.

### Study sample

The main purpose of qualitative data collection was to gain a deeper understanding on the meaning and value of music for people with dementia. In order to build a holistic picture, four groups – family carers, care home staff, music therapists and people with dementia themselves (care home residents and day hospital clients living in the community) – were identified, and the researchers began to organise separate focus groups and interviews for the four groups.

Two National Health Service care homes (home A and home B) that are primarily for people at mid to late stages of dementia agreed to participate as the main research sites. Most care home residents needed assistance in all aspects of their activities of daily living. Although the residents’ verbal communication skills were greatly affected by their dementia, many could still express their opinions. Due to the severity of their dementia, the researchers decided to combine care home residents’ and family carers’ focus groups and interviews so that families could provide supplementary information when needed.

Focus groups with care home staff were organised in negotiation with the care home managers in order to minimise disruption to the day-to-day running of the homes. Music therapy clinical work has been implemented for 3–4 years in both homes, and all staff and the majority of family carers had a general knowledge of what music therapy offered, even though the understanding of the therapeutic process varied between individuals.

Day hospital clients were at earlier stages of dementia, lived independently in the community with support and were able to articulate their opinions and provide their own consent. The interviews with day hospital clients were conducted independently from care home residents.

Music therapists were recruited through the professional network of the lead researcher (OM). The inclusion criteria for music therapists were (1) they were qualified from a recognised postgraduate music therapy programme, and (2) had a minimum of 4 years’ experience of working with clients with dementia.

Ethics approval from the National Research Ethics Service (NRES Committee London East, REC reference 11/LO/0596) was obtained.

### Focus groups and interviews

Focus groups are often preferred to individual interviews as group discussions generate ideas and encourage interactions between participants; however, a larger number of individual interviews were held than initially anticipated. This was mainly due to time constraints; for example, work and family commitments of participants and the limited time allowance for the project, but also due to clinical reasons. OM observed visible frustration in some of the residents when they were invited to group meetings (e.g., their ward round) but did not fully follow what was happening in the meetings. Focus group participation was impractical for some of the residents. Thus, although all the residents in the two homes were invited to focus groups, more individual interviews were conducted in the end. Written consent or assessment forms were completed by all the study participants. Focus groups and interviews were audio recorded whenever possible. When recording was not possible, OM made notes immediately after each interview.

Focus groups and interviews aimed to investigate the meanings and experiences of music for people with dementia and observed effects of music. The key questions asked were:

What does music mean to you? What do you think of your music therapy/music activities? In what way is music important to you? (People with dementia)

What changes and responses do you observe in your families/clients following music therapy or music activities? How do you know if music is meaningful to the person? (Families, staff and therapists)

### Initial analysis of qualitative data

The recordings and interview notes were analysed utilising the general inductive approach (Thomas, 2006) guided by specific evaluation objectives. This allowed the researchers to focus on the extraction of recurring key phrases and common themes that were potentially relevant to an outcome measure development. Additionally, the long-table approach (Kruger & Casey, 2000) was applied in order to increase transparency of the analysis process. Transcription cards of key comments were produced. OM and MO examined the initial extraction of the key comments several times to identify emerging themes. The results of the initial open coding thematic analysis were then discussed with HMR to identify relevance to music therapy clinical work and therapeutic goals.

### The development of Music in Dementia Assessment Scales (MiDAS) version 1

The results of the initial analyses were presented to professors and PhD researchers at the Doctoral Programme in Music Therapy, Aalborg University, to gain further feedback. In addition, an expert consultation with Dr Chris Gilleard was conducted when the emerging themes and the key comments were explored further and potential scale types and scheme for scale item development were discussed. The transcription cards were reviewed again and the key themes were reduced further to develop scale items. The MiDAS version 1 was produced.

### Field-testing of the MiDAS version 1 in practice

The MiDAS version 1 was field-tested by a music therapist and staff in a care home for 6 weeks. The therapist and staff were asked to complete weekly MiDAS ratings. OM and MO examined completed forms to identify strengths and weaknesses of the pilot scale. At the end of field-testing, feedback from the therapist and selected care home staff were obtained, and an evaluation of MiDAS was conducted.

### Refinement of MiDAS version 1 and the production of MiDAS version 2

MiDAS version 1 was presented at the 7th Nordic Music Therapy Congress in Finland to gain feedback from music therapy experts. Three dementia specialists music therapists in the UK and in USA were also asked to make comments on MiDAS to achieve consensus on clinical relevance of the scale items. The outcome of the field-testing, feedback from music therapists and care home staff, and feedback from the conference presentation were incorporated to review the MiDAS version 1. OM, MO and HMR discussed the review results to achieve consensus. MiDAS version 2 was produced.

## Results

### Focus groups and interviews

A total of 6 focus groups and 19 interviews were conducted. The participants (total *n* = 53) comprised care home residents with dementia (12), family carers (15), care home staff (14), day hospital clients with dementia (4) and music therapists (8). The lengths of the interviews and the focus groups varied: from 15 minutes (day hospital client) to an hour and half interview with one family. An average focus group with staff and interviews with residents and families lasted 1 hour; an average interview with music therapists lasted 1 hour and 15 minutes.

### Analysis of qualitative data

OM listened to the recordings of the interviews and focus groups several times and reviewed the interviews notes to familiarise herself with the data. Since the purpose of the analysis was to extract potential key concepts for the measures development, the recordings were not transcribed verbatim. Instead, OM focused on identifying descriptions of personal experiences and meanings of music, observed effects of music and other re-occurring themes and key phrases. The initial transcriptions were discussed with MO and HMR. Further data reduction using the general inductive approach and the long-table approach yielded 270 transcription cards of the key comments. Common themes, for example, increased alertness and interest in people with dementia following music intervention, relationships between music and personal identity, effects of music on mood enhancement, social aspects of group music making/music listening, began to emerge.

### Peer and expert consultations

The results of the initial open coding thematic analysis were discussed with the professors and PhD researchers at the Doctoral Programme in Music Therapy, Aalborg University. Key concepts of musical interactions in music therapy such as attention, awareness, turn-taking and musical engagement were highlighted by the group. However, no consensus on scale items was achieved at this point, and the challenges and limitations of quantifying meaningful musical experiences were discussed.

An expert consultation with Dr Gilleard was held. Types of scales were discussed, and it was decided that Visual Analogue Scales (VAS) without anchor points, rather than Likert scales, might be more suitable for our purpose. VAS is commonly used as a self-rating scale to measure pain (e.g., Huskisson, 1974) and assess mood (e.g., Folstein & Luria, 1973). It is a validated measurement tool, sensitive to change (Wewers & Lowe, 1990) and suitable to measure subjective phenomena such as the intensity of a sensation or experience (Gift, 1989).

The challenge of reducing highly individual musical experiences to several scale items was also discussed. Dr Gilleard suggested OM to investigate scale items in the Multiple Affect Adjective Checklist (MAACL, Zuckerman & Lubin, 1965): a self-rating measure of anxiety, depression and hostility assessing the subject’s current affect. The MAACL includes both negative affect (e.g., nervous, sad, angry) and positive affect items (e.g., happy, enthusiastic, friendly). While the inclusion of both negative and positive effects were considered useful for the new measure, many MAACL items contained internal emotional experiences that are not easily observable in people with moderate to severe dementia. Although not all the 90 items were applicable to an observational measure for people with dementia, the MAACL provided a useful reference point.

### Identification of the key themes and conceptual development for VAS

Further exploration of the qualitative data followed. The emerged five key areas of the impact of music on people with dementia are described below. The names of the participants quoted below have been changed.

#### VAS item Interest

Personally meaningful music often caught people’s attention immediately. Families, staff and therapists reported notable changes in the body postures and increased alertness in the facial expressions of residents who at other times showed little interest in other stimuli. Music was highlighted as one of the few mediums to which most people could still access. Families particularly emphasised the importance of mental stimulation for people with dementia, which was also reflected in the comments from day hospital clients.

Medication is horrible, side effects, makes you sleepy, that’s what upsets me… [but] music focuses you… different from reading paper… I get carried away [when singing]… so stimulating mind, you don’t want to stop. (Ronald: day hospital client)

Resident A, he just lies in his bed now… [music was] comforting, some stimulus, whatever left there… he was so obviously listening. *How did you know?* His face. (Thomas: staff)

#### VAS item Response

Therapists and staff discussed how the residents who usually showed a limited awareness of, or an interest in, other people often responded to other residents during music therapy or interact with staff during sing-alongs. Signs of awareness and degrees of responses were sometimes very subtle. Nevertheless, head turning, longer eye-contact or the changes in the emotional qualities of vocal responses were all considered important indicators of responses when working with people at late stages of dementia.

The way they vocalise… often shows therapy process. One client used to shout “no, no, shut up” but now, she vocalise… fast and sounds angry… but more interactive and aware of me… emotional quality in her voice… her facial expression is very different now. (Fiona: therapist)

… before their music therapy group, G is often withdrawn, J wanders around the corridor, M is stuck in her wheelchair… no grouping… [but during a session], a real sense of group interaction and humour… when M sang, G said “I give you £2 for that”…. awareness of music, awareness of others in the group. (Valerie: therapist)

#### VAS item Initiation

Emotionally meaningful musical experiences often encouraged people to take more initiatives. People often requested songs they wanted to sing, or began to explore new instruments as they became more confident. Music often triggered memories and people were often willing to share their life stories or reminisce. Retained memories of song lyrics despite the progression of dementia were frequently reported.

I think music always brings back their memory, [they] remember old songs… you can see the changes… Resident E starts dancing… once he told me ‘that’s not the way you dance’ so he started teaching me how to dance. (Sophie: staff)

I don’t do much when I am at home… maybe watch TV, I feel lonely, but music therapy is living [experience]… we learn how to play instruments, xylophone, learn rhythm, how to play together. (Ahmed: day hospital client)

#### VAS item Involvement

Music was a flexible medium and allowed different levels of participation according to individual needs, thus encouraging people to get involved at their own pace and sustain their engagement during the activity. Families and staff highlighted the importance of live music for care home residents and discussed how they valued social aspects of group music activities.

My mother danced all the way through [during live music in care home]. One resident started swaying to music…one of them couldn’t speak but started dancing with another resident. It was wonderful to see the changes… people are often so isolated, but everyone just blossomed… music transformed all of them. (Laura: family)

I am 82 now. I don’t always get involved, but… [he is now involved in music making]. I’m old now, but it [playing the drum] gives me a bit of life. I enjoy music therapy group. (Trevor: day hospital client)

#### VAS item Enjoyment

Effects of music on mood were frequently discussed. Day hospital clients emphasised the enjoyment of shared group music making with other group members. Staff frequently reported the calming and relaxing effects of music on agitated or restless residents. People with dementia frequently used the word “happy” when describing their experience of music: for instance, “music makes me happy”, “I feel happier after my music therapy group”.

We play at-random [in music therapy group], but I enjoy it very much…sometimes [people have] different opinions, but we play together, sometimes difficult, each one of us is different… country, knowledge… not professional but I enjoy everything. (William: day hospital client)

Music can help to build something positive… music can help them relax…closing their eyes, tapping their feet, listening to music and just enjoying themselves… I think it’s for all of us [residents and staff]. (Louise: staff)

These five components needed to be captured in the MiDAS scale items but had to be reduced further to several key words that were comprehensive to a wide range of raters. The researchers tried to identify observable mood and behaviours for each component that could represent the five core components. Some of the key comments, for example, “emotionally meaningful experience”, music making people “happy”, were not necessarily observable, thus the researchers had to accept that not all the key aspects would be covered by the VAS items. Thus, the five components – *Interest, Response, Initiation, Involvement* and *Enjoyment* – were designated as the five VAS items.

### Construction of MiDAS version 1

MiDAS version 1 consisted of the five VAS items and a supplementary checklist. Each VAS item comprised a 100 mm line without intervals, with the two extremes of the scale labelled as “none at all” and “highest”. The supplementary checklist was added to provide information on the common presentations (behaviour/mood) of a client that the study participants frequently discussed but were not covered by the five VAS items. This checklist asked if major reactions from the resident were observed in eight areas: vocal agitation, physical aggression, withdrawal, tearfulness, anxiety, laughter, enthusiasm and/or relaxed mood. A space for the rater’s comment was also provided. Two forms, one for music therapists and the other for care home staff, were produced. The five VAS items – Interest, Response, Initiation, Involvement and Enjoyment – and the checklist items were identical in both forms, but the clinical examples provided for each VAS were different.

### Rating procedure

Staff MiDAS form asked a staff member with sufficient knowledge of the resident to complete rating A 15 minutes before the resident’s music therapy session. The staff member was to observe the resident for 5 minutes to decide the average rating for each of the five VAS items. The same staff member observed the resident for further 5 minutes and completed rating B 2 hours after the resident’s music therapy. The music therapist was asked to complete rating 1 and rating 2 immediately after the session. The therapist’s rating 1 applied to the average presentation of the client during the first 5 minutes of the session, while rating 2 applied to the clinically most meaningful 5 minutes during the entire session. Thus, a total of four MiDAS forms were completed per resident on the day of music therapy.

The researchers initially anticipated the staff rating B might be conducted a day after a music therapy session in order to assess the short- to medium-term effect. However, the interviewees and focus group participants mostly focused on the immediate effects of music during music listening or playing instruments and the effects on the people’s mood on the same day. The qualitative data did not provide sufficient information that it would be effective to conduct the follow-up ratings beyond the same day. Thus, MiDAS was constructed as a *Same-Day-Scale*.

### Outcome of the field-testing of MiDAS version 1

Eighty-five MiDAS forms were completed by the music therapist and selected care home staff during the field-testing of MiDAS version 1. All the MiDAS forms were fully completed and no floor-ceiling effect was found. All the raters confirmed the form was easy to use and the scale items were clinically appropriate.

However, it was noted that several staff members struggled to grasp the concept of VAS because it required the rater to take a moment to stop and reflect what would be the optimum level for that individual. Some staff fed back that this was not an easy task especially when they were under time pressure to complete daily tasks such as monitoring adequate food and fluid intake for residents and ensuring the safety of their physical environment.

Specifying the exact timing for staff ratings proved impractical and ineffective. The weekly music therapy group took place after breakfast. When the staff were completing *rating A* before the session, many residents were back in their room or sitting quietly in front of TV, thus their MiDAS scores were very low even though they might have been more interactive during the breakfast. The music therapist reported some residents were extremely conscious of being observed by staff members. Time specification for staff *rating B* after the music therapy session was not always practical due to work commitments.

### Evaluation of MiDAS version 1: changes on Staff MiDAS form and the rating procedure

The following modifications were made to the pilot MiDAS form. Staff *ratings A* and *B* were changed to *before* and *after* to make ratings clearer in relation to the timing of music therapy. Staff raters were asked to *take a moment and reflect on the resident’s behaviour and mood today and decide the average rating for each scale* and the request for observation was withdrawn. The two staff forms were still to be completed on the same day, but exact timings of the ratings were no longer specified. The revised form requested that a *before* form to be completed before the resident’s music therapy session on the day and an *after* form to be completed on the same day, ideally a few hours after the music therapy session, to gain an overall picture of the resident on the day.

### Evaluation of MiDAS version 1: changes on therapist MiDAS form

Therapist *ratings 1* and *2* were changed to *beginning* and *during*. Explanations on the presentation of the resident “during the first five minutes” and “during the best five minutes” remained unchanged. The rating procedure remained the same.

### Review of the MiDAS items

Potential difficulties in the conceptual understandings of the two VAS items, *Initiation* and *Involvement,* were pointed out by music therapists and staff. Possible alternative wordings such as communication (instead of Initiation) and engagement (instead of Involvement) were suggested by music therapists, but no consensus was achieved. At the end, the researchers decided to keep the five VAS items the same. Clinical examples for each VAS were reviewed. Key behavioural and mood signs to look for were highlighted and shorter question forms were applied in an attempt to make the rater stop and reflect before making a mark on the VAS.

The eight items on the supplementary checklist were also reviewed. OM reviewed the completed MiDAS forms and the 270 transcription cards again. Six items (vocal agitation, physical aggression, withdrawal, tearfulness, laughter and relaxed mood) were kept, two items (anxiety and enthusiasm) that were rarely used during the field-testing were deleted, and two new items (attentive/interested, cheerful/smiling), the presentations frequently mentioned in the transcription cards, were added. At the end, four positive reactions (attentive/interested, cheerful/smiling, relaxed mood, laughing) and four negative reactions (vocal agitation, physical aggression, withdrawal, tearful) replaced the original items (three positive reactions and five negative reactions).

### Production of MiDAS version 2

Finally, the background information on MiDAS and the instructions for rating MiDAS on the pilot form were simplified to keep the raters’ focus on the scales themselves. The form was re-formatted to optimise visual clarity. A separate one-page guidance sheet was produced for a new MiDAS rater.

The final version of MiDAS is shown in Appendix.

## Discussion

MiDAS is the first dementia-specific quantitative music therapy outcome measure developed rigorously from qualitative data utilising focus groups, the long-table approach, peer and expert consultations, field-testing and the evaluation of the scale items. This gives MiDAS a strong content validity.

MiDAS was not designed to model an infant and parent interaction, but the five VAS items – *Interest, Response, Initiation, Involvement* and *Enjoyment* – show some resemblance to such interaction. If an infant shows an *interest* in what his/her parent is initiating, he/she may *respond* to that stimuli. If the stimuli or their interaction is meaningful, the infant may *initiate* an activity or communication in return. When this interaction is sustained, both parties become more *involved* in the activity, which may give the infant an *enjoyable* experience.

These similarities may be linked to the nature of basic musical interaction. Trevarthen & Malloch (2000) stated that “music making is a human activity that communicates motives” and explained that early parent–infant communication as “innate musicality” represents “outward signs of human communication”. Kitwood (1993) also argued for the importance of recognising and supporting the “communicative attempt” of a person with dementia and analysed the key stages of the “communicative act” that involved recognition of an initial gesture and response, sustaining the interaction and “holding” the emotional experience. Kitwood also draws a parallel to the “psychology of infancy and early childhood”. The fact that qualitative data provided by the focus groups participants and interviews were sufficient enough for the researchers to recognise these key elements for MiDAS development also supports the content validity of MiDAS.

MiDAS was not developed for a specific music therapy approach. However, the majority of music therapists interviewed used an active music therapy model, and staff and families also highlighted the importance of active music making, singing and dancing with music. Given the nature of MiDAS that focuses on a person’s visible reaction to musical stimuli and musical interactions with others, it is possible MiDAS may be more useful for an active music therapy model. It may be worthwhile in the future to further explore the strengths and limitations of MiDAS.

The application of VAS may be one of the strengths of MiDAS. VAS scales are based on individual optimal levels (the “best score” that that individual can achieve) rather than uniform, predetermined set of scores. VAS is particularly advantageous to assess the optimum level of a person with dementia since the optimum level may not only differ between individuals but may also shift as the dementia progresses. Our recent study explored the psychometric properties of MiDAS (McDermott, Orgeta, Ridder & Orrell, 2014). A total of 629 forms were completed (mean = 238.87, SD = 136.38). The statistical analysis revealed that the distribution of the MiDAS scores covered the full range and there were no floor and ceiling effects. MiDAS is not intended to replace existing outcome measures such as the NPI, the CMAI and quality of life scales. Instead, MiDAS may help identify how far participants engage with music therapy and this may help to explain who benefits in terms of other outcomes. Thus, the explanatory power of MiDAS may become useful in clinical trials, non-controlled longitudinal studies and during routine clinical evaluations.

Evaluation of the changes in the MiDAS scores over the course of music therapy may offer insight into the therapy process and help illuminate how the individual benefitted from music therapy. When MiDAS is used in conjunction with other measures (e.g., NPI, quality of life measures, cognitive tests), it may be useful to examine whether MiDAS scores correlate with the changes in neuropsychiatric symptoms, cognitive tests or quality of life. This will help evaluating whether music therapy impacts on other areas of the person’s life. Music therapists often report increased musical responsiveness in people with dementia even when their cognitive and physical functions become depleted. Music therapy-specific measures may identify subtle, yet potentially clinically important changes in musical and interpersonal relationships and increased social engagement that other measures may not be sensitive enough to pick up. Further analysis of the qualitative data revealed that (1) the effects of music for people with dementia go beyond the reduction of behavioural and psychological symptoms, (2) individual preference of music is closely linked to personal identity and personal history and (3) sustaining “here and now” musical and interpersonal connectedness help value the uniqueness of an individual and maintain the quality of his/her life (McDermott, Orrell & Ridder, 2014).

### Limitations of the study

There are some limitations and methodological issues in this study. One of the limitations of MiDAS is that it scores high only when a person is visibly active. Since MiDAS focuses on observable responses/non-responses from a resident, VAS items should not be scored when a resident was asleep or appeared asleep for most of the time during the observation period. Instead, a rater is asked to provide relevant information (qualitative data) under “any comments”. This is because it is crucial to differentiate the lack of observed effects of music/responses to music from the lack of observable behaviour because a resident was asleep. The raters frequently pointed out during the field-testing that falling asleep or closing their eyes are common behaviours for many care home residents. It should be noted that MiDAS focuses on observable effects of music.

Although the application of VAS may be one of the strengths of MiDAS, the use of VAS also proved to be a challenge, and the inconsistency of the MiDAS raters is one of the limitations of this study. Staff raters sometimes found the concept of the VAS too difficult to grasp, and it was also not possible to have the same staff rater for the same client every week due to the nature of care home shift work. Although the raters were requested to score what they observed and to keep their interpretation to a minimum, a proxy measure inevitably involves personal interpretation. The music therapist that field-tested the MiDAS and regularly assisted staff raters to complete MiDAS forms questioned the validity of some staff forms that were completed in haste and without much reflection. The importance of training for new MiDAS raters and the need for ongoing monitoring to ensure the validity of observational VAS scales cannot be underestimated.

Since MiDAS evaluates the observable changes during the course of music therapy, it is not suitable to use after the intervention has ceased. Therefore, the potential longer-term effects of music therapy need to be evaluated through other validated outcome measures. If MiDAS is used in a RCT, staff raters should be blind to treatment allocations, which can be a challenge in a care home environment.

Finally, quantitative observational outcome measures have their limitations. It should be noted that MiDAS was not designed to cover all the key components of music therapy with people with dementia. One music therapist commented that the VAS item “levels of enjoyment” did not necessarily represent the most important aspect of music therapy and that the term “enjoyment” can enforce misperception of some staff who regard music therapy more as an entertainment where “patients have fun”. An alternative name “levels of emotional engagement” was suggested. The music therapy process certainly involves a whole spectrum of emotional engagement including expressions of anger and the sense of loss. However, not all “emotional engagement” is observable and rating “emotional engagement” as a VAS item can be too open to personal interpretations of the rater. Since MiDAS focuses on capturing what people with dementia value in music, “enjoyment”, which was frequently named as a significant musical experience by the study participants and a relatively observable emotional response, was kept as a VAS item. The fact that other emotional responses need to be recorded as qualitative data on the MiDAS form does not mean these responses are any less important, but this highlights the challenge and the limitation of quantifying meaningful emotional experiences.

### Future research

Further refinement may include an exploration of weighting VAS items. The current MiDAS does not differentiate the weighting between the five VAS items, but it is possible some items may be regarded clinically more important than others.

A possible future project includes the development of a self-rating version of MiDAS where people with dementia at earlier stages of dementia can complete themselves and a family-carer version to compare the scores of the self-rating MiDAS. The development of self-rating MiDAS may also allow adding another VAS item that relates to more internal, personally meaningful experiences of music or musical identity. Our recent review on music therapy in dementia (McDermott et al., 2013) identified the need for high-quality studies with people with mild to moderate dementia. Therefore, self-rating MiDAS may become useful as a secondary outcome measure. Future music therapy studies in dementia would benefit most from a mixed methods approach that include qualitative feedback from staff and music therapists focusing on the impact of the music on the mental health and the well-being of the person that is unique to each individual.

## Conclusion

This study has demonstrated the robust development procedure for MiDAS and its good qualitative validity based on a consensus process and feedback from a diversity of stakeholders. A further study (McDermott, Orgeta, et al., 2014) has shown MiDAS to have adequate psychometric properties in terms of reliability, internal consistency, concurrent validity and construct validity. Despite the study limitations, the outcome of this study demonstrates it is possible to design and develop a rigorous quantitative outcome measure from the qualitative data that explored the value and the meanings of music for people with dementia. It is hoped that the study highlights the importance of listening to the voices of people with dementia whose values may not always be incorporated in research.

## Appendix

Not to scale. When used for clinical and research purposes, the MiDAS form should be downloaded from DOI: 10.1080/08098131.2014.907333

## References

[CIT0001] Ashida S. (2000). The effect of reminiscence music therapy sessions on changes in depressive symptoms in elderly persons with dementia. *Journal of Music Therapy*.

[CIT0002] Brotons M., Marti P. (2003). Music therapy with Alzheimer’s patients and their family caregivers: A pilot project. *Journal of Music Therapy*.

[CIT0003] Cohen-Mansfield J. (1986). Agitated behaviors in the elderly. II. Preliminary results in the cognitively deteriorated. *Journal of the American Geriatrics Society*.

[CIT0004] Creswell J., Plano Clark V. (2007). Introducing a mixed methods study. J. Creswell & V. Plano Clark (Eds.), *Designing and conducting mixed methods research*.

[CIT0005] Cummings J. L., Mega M., Gray K., Rosenberg-Thompson S., Carusi D. A., Gornbein J. (1997). The neuropsychiatric inventory: Comprehensive assessment of psychopathology in dementia. *Neurology*.

[CIT0006] Folstein M., Luria R. (1973). Reliability, validity, and clinical application of the visual analogue mood scale. *Psychological Medicine*.

[CIT0007] Gift A. (1989). Visual analogue scales: Measurement of subjective phenomena. *Nursing Research*.

[CIT0008] Glynn N. J. (1992). The music therapy assessment tool in Alzheimer’s patients. *Journal of Gerontological Nursing*.

[CIT0009] Huskisson E., Hart (1974). Pain: Mechanism and measurement. *Chronic pain*.

[CIT0010] Kitwood T. (1993). Towards a theory of dementia care: The interpersonal process. *Ageing and Society*.

[CIT0011] Kruger R., Casey M. (2000). *Focus groups. A practical guide for applied research*.

[CIT0012] Ledger A., Baker F. (2007). An investigation of long-term effects of group music therapy on agitation levels of people with Alzheimer’s disease. *Aging & Mental Health*.

[CIT0013] (1995). The use of music performance tasks in the assessment of cognitive functioning among older adults with dementia. *Journal of Music Therapy*, *32*(3), 137–151..

[CIT0014] Livingston G., Johnston K., Katona C., Paton J., Lyketos C. (2005). Systematic review of psychological approaches to the management of neuropsychiatric symptoms of dementia. *American Journal of Psychiatry*.

[CIT0015] McDermott O., Crellin N., Ridder H. M., Orrell M. (2013). Music therapy in dementia: A narrative synthesis systematic review. *International Journal of Geriatric Psychiatry*.

[CIT0016] McDermott O., Orgeta V., Ridder H. M., Orrell M. (2014). A preliminary psychometric evaluation of music in dementia assessment scales (MiDAS). *International Psychogeriatrics*.

[CIT0017] McDermott O., Orrell M., Ridder H. M. (2014). The importance of music for people with dementia: The perspectives of people with dementia, family carers, staff and music therapists. *Aging & Mental Health*.

[CIT0018] Raglio A., Bellelli G., Traficante D., Gianotti M., Ubezio M. C., Gentile S., Trabucchi M. (2010). Efficacy of music therapy treatment based on cycles of sessions: A randomised controlled trial. *Aging & Mental Health*.

[CIT0019] Raglio A., Bellelli G., Traficante D., Gianotti M., Ubezio M. C., Villani D., Trabucchi M. (2008). Efficacy of music therapy in the treatment of behavioral and psychiatric symptoms of dementia. *Alzheimer Disease and Associated Disorders*.

[CIT0020] Ridder H. M. (2013). Mixed-methods research. *International dictionary of music therapy*.

[CIT0021] Svansdottir H., Snaedal J. (2006). Music therapy in moderate and severe dementia of Alzheimer’s type: A case-control study. *International Psychogeriatrics*.

[CIT0022] Thomas D. (2006). A general inductive approach for analyzing qualitative evaluation data. *American Journal of Evaluation*.

[CIT0023] Trevarthen C., Malloch S. (2000). The dance of wellbeing: Defining the musical therapeutic effect. *Nordic Journal of Music Therapy*.

[CIT0024] Vink A., Bruinsma M., Scholten R. (2013a). Music therapy for people with dementia (Review). *Cochrane Database of Systematic Reviews*.

[CIT0025] Vink A., Zuidersma M., Boersma F., de Jonge P., Zuidema S., Slaets J. (2013b). The effect of music therapy compared with general recreational activities in reducing agitation in people with dementia: A randomised controlled trial. *International Journal of Geriatric Psychiatry*.

[CIT0026] Wewers M., Lowe N. (1990). A critical review of visual analogue scales in the measurement of clinical phenomena. *Research in Nursing & Health*.

[CIT0027] York E. (2000). A test-retest reliability study of the residual music skills test. *Psychology of Music*.

[CIT0028] Zuckerman M., Lubin B. (1965). *Manual for the multiple affect adjective check list*.

